# Antisense-mediated isoform switching of steroid receptor coactivator-1 in the central nucleus of the amygdala of the mouse brain

**DOI:** 10.1186/1471-2202-14-5

**Published:** 2013-01-07

**Authors:** Ioannis Zalachoras, Gwendolynn Grootaers, Lisa TCM van Weert, Yves Aubert, Suzanne R de Kreij, Nicole A Datson, Willeke MC van Roon-Mom, Annemieke Aartsma-Rus, Onno C Meijer

**Affiliations:** 1 Division of Medical Pharmacology, Leiden/Amsterdam Center for Drug Research, Leiden University/Leiden University Medical Center, Leiden, The Netherlands; 2 Department of Endocrinology, Leiden University Medical Center, Albinusdreef 2, Building 1, Leiden, 2333 ZA, The Netherlands; 3 Department of Human Genetics, Leiden University Medical Center, Leiden, The Netherlands; 4 Postzone C4-R, P.O. Box 9600, Leiden, 2300 RC, The Netherlands

**Keywords:** Steroid receptor coactivator-1, Antisense oligonucleotide, Exon skipping, Glucocorticoid receptor, Brain

## Abstract

**Background:**

Antisense oligonucleotide (AON)-mediated exon skipping is a powerful tool to manipulate gene expression. In the present study we investigated the potential of exon skipping by local injection in the central nucleus of the amygdala (CeA) of the mouse brain. As proof of principle we targeted the splicing of steroid receptor coactivator-1 (SRC-1), a protein involved in nuclear receptor function. This nuclear receptor coregulator exists in two splice variants (SRC-1a and SRC-1e) which display differential distribution and opposing activities in the brain, and whose mRNAs differ in a single SRC-1e specific exon.

**Methods:**

For proof of principle of feasibility, we used immunofluorescent stainings to study uptake by different cell types, translocation to the nucleus and potential immunostimulatory effects at different time points after a local injection in the CeA of the mouse brain of a control AON targeting human dystrophin with no targets in the murine brain. To evaluate efficacy we designed an AON targeting the SRC-1e-specific exon and with qPCR analysis we measured the expression ratio of the two splice variants.

**Results:**

We found that AONs were taken up by corticotropin releasing hormone expressing neurons and other cells in the CeA, and translocated into the cell nucleus. Immune responses after AON injection were comparable to those after sterile saline injection. A successful shift of the naturally occurring SRC-1a:SRC-1e expression ratio in favor of SRC-1a was observed, without changes in total SRC-1 expression.

**Conclusions:**

We provide a proof of concept for local neuropharmacological use of exon skipping by manipulating the expression ratio of the two splice variants of SRC-1, which may be used to study nuclear receptor function in specific brain circuits. We established that exon skipping after local injection in the brain is a versatile and useful tool for the manipulation of splice variants for numerous genes that are relevant for brain function.

## Background

Alternative splicing in the brain has gained significant attention recently and may be important for a vast number of processes [1] such as synaptic function [2] and learning and memory [1]. Examples of alternatively spliced genes include the D2 receptor gene [3], the corticotropin releasing hormone (CRH) receptor genes [4] and the cannabinoid receptor genes [5,6]. A limitation to the study of the roles of the various splice variants in brain function is that very often specific ligands or inhibitors are lacking. Furthermore, transgenic approaches may be both costly and time consuming, and/or depend on viral delivery which may induce immune responses [7].

Single stranded DNA or RNA antisense oligonucleotides (AONs) that target RNA transcripts can be used to manipulate gene expression in different manners. DNA:RNA or RNA:RNA hybrids can be cleaved by RNase H resulting in knockdown of gene expression. A similar effect can be achieved via steric hindrance of the ribosomal complex by an AON resulting in mRNA translation arrest and blocking of protein expression [8]. A third mechanism involves the hybridization of an AON to intronic/exonic inclusion sequences of primary RNA transcripts, thus rendering specific exons inaccessible to the splicing machinery and leading to skipping of the exon [9]. In a similar fashion, AONs can hybridize to intronic/exonic exon exclusion sequences and result in inclusion of target exons [7,10].

To date, modulation of splicing by AONs has been used as a potential treatment approach for several diseases, including Duchenne muscular dystrophy (DMD) and models of spinal muscular atrophy (SMA) [10-12]. Effective protein restoration in DMD via exon skipping has been shown in patient derived cell cultures, animal models, (reviewed in [9] and even in clinical trials [13,14]. Similar results have been obtained in SMA via the related mechanism of exon inclusion [10,15-17].

**Figure 1:**
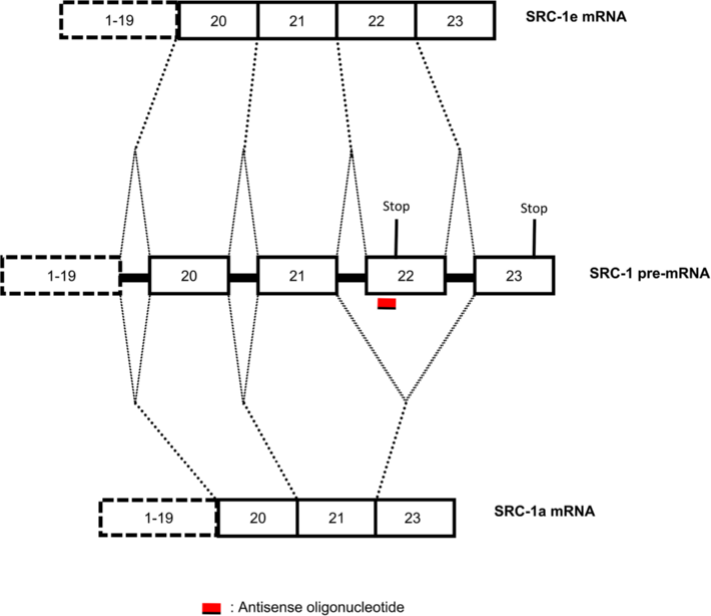
**Schematic representation of the mRNA of the two SRC-1 splice variants.** Boxes represent exons and thicker full lines represent introns. Dashed lines indicate possible splicing events. The approximate position of stop codons is also marked. If exon 22 is included, SRC-1e is expressed. Exon 22 contains an earlier stop codon, therefore SRC-1e protein is shorter than SRC-1a. AONs targeting exon 22 can render it inaccessible to the splicing machinery and therefore, shift the expression of SRC-1 towards SRC-1a. (Adapted from Kalkhoven et al. [23]).

Despite the potential of splicing modulation, AONs have been used in an experimental setting mainly to induce knockdown of gene expression [18-20], while antisense-mediated modulation of splicing has not been used widely as a research tool in the brain. One of the obstacles preventing their more widespread application in the central nervous system (CNS) is their inability to cross the blood-brain-barrier of adult animals [21]. Nevertheless, when AONs are applied directly to the CNS via intracerebroventricular (ICV) or intrathecal administration, the results show considerable potential [10,12,21] and long-lasting effects [10,21]. In this study we evaluated the efficacy and occurrence of immune-related side effects after a single local AON injection in the central amygdala of the mouse brain. As proof of principle, we targeted steroid receptor coactivator-1 (SRC-1), a gene that codes for two splice variants, SRC-1a and SRC-1e, which only differ in one exon (Figure 1; [22,23]). SRC-1 can act as a coregulator of glucocorticoid receptor (GR) dependent transcription [24], as well as of other nuclear receptors [25]. The SRC-1 splice variants show differential activity and distribution in the brain [26]. The splice variants have been shown to exert opposite effects on the GR-mediated regulation of the *crh* gene [27].

We targeted exon 22 of the *SRC-1* gene (Figure 1) using AONs, examined their cellular uptake by different cell types, exon skipping efficacy over time and potential immunostimulatory effects. For cellular uptake and potential immunostimulatory effects we used an AON targeting human dystrophin that has no known targets in the murine genome, in order to investigate the target-independent physico-chemical properties of 2-O’-methyl modified phosphorothioate oligonucleotides. Our results showed adequate uptake by cells in the CeA and translocation into the cell nucleus, combined with detectable isoform switching until at least 7 days after a single injection and a practically complete lack of immunostimulatory effects compared with vehicle injection.

## Results

### Cellular uptake

**Figure 2:**
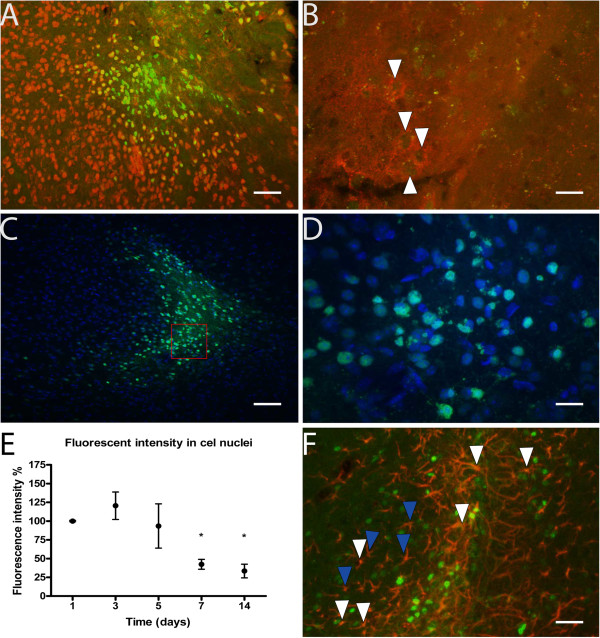
**AON uptake by different cell types and nuclear localization in the central amygdala (CeA). A**. Uptake of AONs by neurons. The green fluorescence of the labeled AONs is colocalized with NeuN (red), a marker of neurons. Scale bar 50 μm **B**. Uptake of AONs by cells expressing CRH. AONs (green) are located in the nuclei of those cells (white arrowheads), surrounded by CRH in the cytoplasm (red). Scale bar 15 μm. **C**, **D**. Localization of AONs (green) in the cell nucleus, colocalized with the nuclear marker Hoechst (blue). Scale bar 50 μm. The area within the red square is magnified in D (scale bar 15 μm). **E**. Fluorescent intensity in the cell nuclei after an injection of AONs. Fluorescence on day 1 was normalized to 100%. One-way ANOVA (F_(4,14)_= 7.845, p<0.01) followed by Dunnett’s post hoc test (all groups compared to the 1 day group) showed a significant decrease of fluorescence after 7 and 14 days (Dunnett’s test p<0.05 in both cases). Between the 7- and 14-day groups there was no further decrease. **F**. Uptake of AONs (green) by GFAP (red) positive astrocytes. Several astrocytes have taken up AONs while others have not. White arrowheads indicate a few examples of astrocytes that took up AONs, whereas blue arrowheads indicate a few examples of those that did not. Scale bar 30 μm.

In order to investigate the cell types and intracellular destination of 2-O’-Methyl phosphorothioate AONs we performed immunofluorescent detection of CRH (which is expressed in the CeA), NeuN and Hoechst after local injection of an AON targeting human dystrophin, which has no known targets in the mouse. Our results showed that fluorescently labeled AONs were taken up by neurons in general, as well as neurons expressing CRH in the CeA, and also translocated into the cell nucleus (Figure 2A-D). Quantification showed that 67.5% ± 2.6 of the cells that had taken up the AONs was NeuN positive. This indicates that the AONs can indeed be taken up by neurons in the brain and translocate to the nucleus where splicing events take place.

### AON detection

In order to determine the stability of AONs in the brain after local injection we measured the intensity of the green fluorescence originating from the fluorophore conjugated to AONs in the brains of animals sacrificed 1, 3, 5, 7 and 14 days after a single injection with an AON targeting human dystrophin. Fluorescence intensity did not differ significantly between 1 and 3 days but subsequently decreased over time to less than 50% in 7 days (Figure 2E). After 7 days, fluorescence intensity remained stable until the last detection time point, 14 days post-injection. In these calculations only green signal colocalized with Hoechst (cell nuclei) was taken into account, thus restricting our analysis to a functionally relevant subcellular compartment.

### Diffusion of AONs

In order to investigate the specific targeting of a selected brain region we measured the diffusion of the AONs around the injection site. Our results indicated a well localized targeting of about 0.1 mm^3^ (Table 1).

**Table 1:** Diffusion of the AONs in the brain in the mediolateral, dorsoventral and anterior-posterior axes

**Measurement**	**Size**	**SEM**
Mean mediolateral diffusion	505	70
Mean dorsoventral diffusion	671	84
Mean anterior-posterior diffusion	350	34
Maximum mediolateral diffusion	903	-
Maximum dorsoventral diffusion	1015	-
Mean Volume	0.11	0.03

Lengths in μm, volumes in mm^3^. Data shown as mean ± SEM.

### Immunostimulatory effects

**Figure 3:**
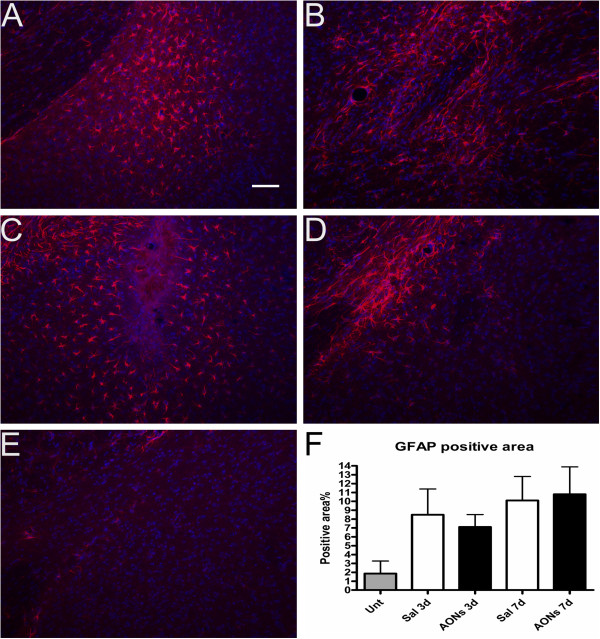
**GFAP immunoreactivity 3 or 7 days after a single injection in the CeA. A**. 3 days after a single saline injection. **B**. 7 days after a single saline injection. **C**. 3 days after a single injection of AONs. **D**. 7 days after a single injection of AONs. **E**. GFAP immunoreactivity in the CeA of an untreated mouse. **F**. Quantification of GFAP immunoreactive area shown as percentage of the total area of visual field. One-way ANOVA followed by Tukey’s post hoc test revealed no significant differences between the respective AON and saline injected animals (one-way ANOVA F_(4,17)_=1.266, p>0.32, N=3-7 animals per group). In conclusion, a single AON injection did not induce stronger astrocytosis than saline. Scale bar 50 μm. Red: GFAP, blue: Hoechst. Green signal (AONs) has been omitted for clarity.

**Figure 4:**
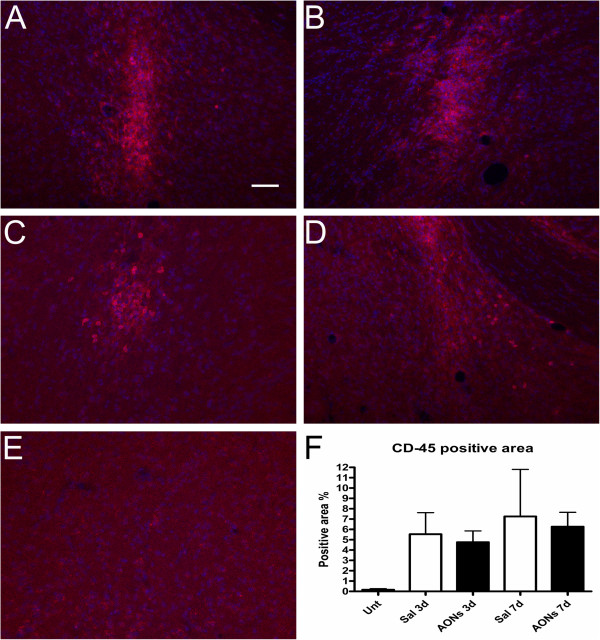
**CD-45 immunoreactivity 3 or 7 days after a single injection in the CeA. A**. 3 days after a single saline injection. **B**. 7 days after a single saline injection. **C**. 3 days after a single injection of AONs. **D**. 7 days after a single injection of AONs. **E**. CD-45 immunoreactivity in the CeA of an untreated mouse. **F**. Quantification of CD-45 immunoreactive area shown as percentage of the total area of visual field. One-way ANOVA followed by Tukey’s post hoc test revealed no significant differences between the respective AON and saline injected animals (one-way ANOVA F_(4,17)_=1.092, p>0.39, N=3-7 animals per group). Quantification of IBA-1 immunoreactive area had similar results (one-way ANOVA F_(4,17)_=1.535, p>0.23, data not shown). In conclusion, a single AON injection did not induce stronger microglia activation than saline. Scale bar 50 μm. Red: CD-45, blue: Hoechst. Green signal (AONs) has been omitted for clarity.

We analyzed two different markers for microglia activation (CD-45 and IBA1) and one marker for astrocytes (GFAP). CD-45 is a marker of activated microglia, whereas IBA-1 is a constitutive marker of microglia [28,29]. We compared AON-injected (with an AON targeting human dystrophin) to saline-injected animals 3 and 7 days after the injections. Moreover, we included an untreated group of animals to assess the effects of the injections. No differences were observed between saline and AON treated animals at either time point (Figures 3,4). AON uptake was also observed in a subset of GFAP positive astrocytes (Figure 2F). Little or no uptake by microglia was observed.

### Isoform switching

**Figure 5:**
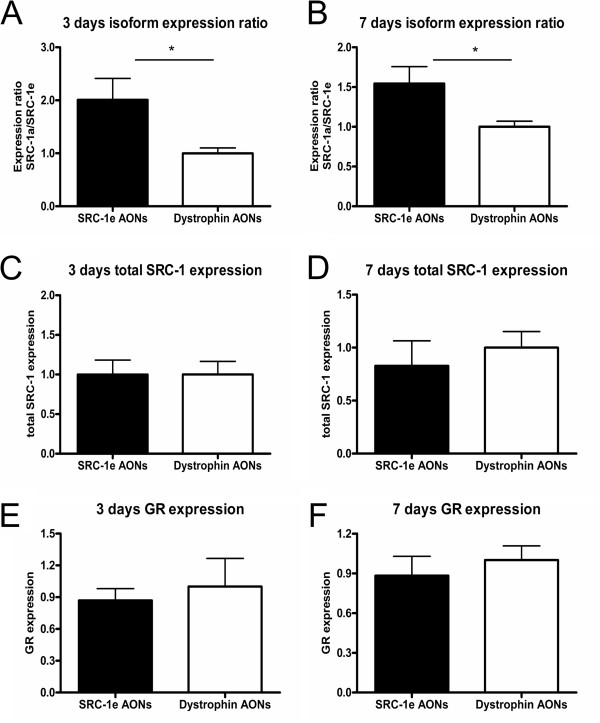
**qPCR analysis of gene expression 3 or 7 days after a single injection. A**. Relative expression of the SRC-a1/SRC-1e 3 ratio days after a single injection of AONs. AON treatment targeting exon 22 of SRC-1e leads to a 2-fold difference of the expression ratio of the two isoforms in favour of SRC-1a (independent t-test, t_(6)_=2.414, p<0.05, n=6-7 per group). **B**. Relative expression of the SRC-1a/SRC-1e ratio 7 days after a single injection of AONs. AON treatment targeting exon 22 of SRC-1e lead to 1.5-fold difference of the expression ratio of the two isoforms in favour of SRC-1a (independent t-test t_(8)_=2.420, p<0.05, n=5 per group). **C**, **D**. Treatment with AON targeting exon-22 of SRC-1e had no effect on total SRC-1 expression compared to control 3 (**C**) or 7 days (**D**) after a single injection (independent t-tests, t_(11)_=0.006, p>0.99 and t_(7)_=1.304, p>0.57 respectively, n=4-7 per group). **E**, **F**. GR expression remained unchanged between animals injected with AON targeting exon 22 of SRC-1e and controls 3 (**E**) or 7 days (**F**) after a single injection (independent t-tests, t_(11)_=0.479 p>0.64 and t_(7)_=0.662, p>0.52, n=4-7 per group).

In order to determine the efficacy of AONs treatment on exon skipping in the brain we used qPCR analysis to measure the expression ratio of the two isoforms in the CeA, 3 and 7 days after a single injection with either an AON against SRC-1e or a control-AON. Three days after the injection the SRC-1a:SRC-1e ratio showed a 2-fold shift in favor of SRC-1a in the group injected with AONs against SRC-1e, in comparison to the control-AON injected group. However, total SRC-1 expression was not different between the groups (Figure 5). Seven days after injection the expression ratio was still significantly higher in the animals injected with AONs against SRC-1e (approximately 1.5-fold higher than their control injected counterparts) without a difference in total SRC-1 expression. As an additional control for specificity, mRNA for GR (which may be one of the target nuclear receptors of SRC-1) was not significantly different between the groups either at the 3- or the 7-days time point. In view of previously reported upregulation of SRC-2 in SRC-1 knockout mice [30], we determined SRC-2 mRNA. We did not find a significant difference between the two groups regarding SRC-2 expression 3 days post injection. SRC-2 expression was 0.7 ± 0.2 for animals injected with AONs targeting SRC-1e and 1.0 ± 0.3 for animals injected with human dystrophin (independent t-test, t_(6)_=0.8511, p>0.42).

## Discussion

In this study, we investigated the efficacy in AON-mediated isoform switching, AON uptake by different cellular types and the putative immunostimulatory effects of AONs, in order to evaluate their potential use as a tool in experimental brain research.

Our results showed that it is possible to alter the expression ratio of the two SRC-1 isoforms with a single injection of AONs targeting exon 22 of the transcript of SRC-1e. Three days after the injection the isoform expression ratio showed a 2-fold increase in favor of SRC-1a, whereas 7 days after a single injection of AONs the respective difference was approximately 1.5-fold in favor of SRC-1a. In order to confirm that this was a genuine effect and was not influenced by downregulation of total SRC-1 we also investigated total SRC-1 expression in the two groups, which was shown to be comparable and not significantly different at both time points. We also investigated the expression of GR to control for possible differences as a consequence of off-target non-homologous binding of the AONs. We selected GR as an additional control, because SRC-1 is involved in GR-dependent pathways. Our results showed that GR mRNA expression is not significantly different between animals injected with either an AON targeting SRC-1e or a control AON 3 or 7 days after a single injection. This finding indicates no difference in non-homologous targeting between the specifically targeted and the control AONs and is also relevant for future experiments attempting to unravel the role of SRC-1 and its isoforms in GR dependent pathways as any effects can be attributed solely to SRC-1 isoform switching. Since SRC-2 has been shown to be upregulated in the absence of SRC-1 during development [30], we investigated its expression 3 days after injection in order to rule out an effect of SRC-1 isoform switching on SRC-2 expression. We did not find SRC-2 upregulation, which is in line with the absence of effects on total SRC-1. Although a larger-scale transcriptome and/or proteome analysis would be necessary to investigate all potential off-target effects of the AONs used in this study [31], our results from total SRC-1, SRC-2 and GR mRNA expression indicate high specificity. In addition, since AONs do not obligatorily interfere with endogenous pathways, unlike siRNAs, they cannot saturate the cellular miRNA machinery [21], thus avoiding a source of off-target effects.

Regarding the effect size of the AONs’ efficacy, it is important to note that the dissected area, particularly at longer distances from the injection site may contain cells that did not take up AONs. For the group treated with AONs against SRC-1e that would mean a dilution of the effect. Therefore, the actual efficacy of exon skipping could well be higher than observed.

The detection of isoform switching 7 days after a single injection of AONs allows animals sufficient time for post-operational recovery and performance of additional experiments, for instance, behavioral experiments. In addition, we were able to detect fluorescence of AONs up to 14 days after injection, which is probably accompanied by isoform switching to some extent, although the decrease of the expression ratio of the two isoforms between 3 and 7 days indicates that the effect size may decrease over time. If longer lasting effects are required, potential solutions may involve higher doses and repeated or continuous administration [21]. Persisting effects have been shown even 6 months after termination of continuous infusion of AONs for 7 days in the ventricles of the brain [10], and even single administration may have long-lasting effects [32].

Astrocytosis and microglia activation may confound any findings in relation to brain function. We found no differences in the immune responses caused by a single injection of AONs or a single injection of sterile saline 3 or 7 days after the injections. The time course of astrocytosis and microgliosis that we observed both in vehicle and AON treated animals was similar to what has been previously reported for saline injections [29]. It is unlikely we have reached a plateau in immune responses with saline, since it has been shown in the past that administration of lipopolysaccharide causes substantially stronger immune responses than saline [29]. Immunostimulatory effects that have been observed in other studies may have been caused by the vehicle used [33], or immune responses elicited by simulation of Toll-Like receptors (TLRs) through the phosphorothioate backbone of the AONs [34]. However, 2-O’-modifications may act as TLR antagonists [35], which may account for the lack of immune responses in our study (in spite of the high local concentrations of AONs), as well as in others [21]. Hua et al., 2010 reported an upregulation of IBA-1 mRNA expression after continuous infusion for 9 days of 2-O’-Methyl modified AONs but not of 2-O’-Methoxyethyl AONs compared to saline. This discrepancy between the current study and the study of Hua et al. may be due to the different experimental setup. The current study used a local single injection of ~1 μg of AONs instead of a continuous ICV administration of 10 μg or more per day for 9 days that induced significant upregulation of IBA-1 in the spinal cord, or 30 μg or more that was necessary to induce significant upregulation of IBA-1 in the brain. Administration of 10 μg of AONs per day was not enough to cause significant IBA-1 upregulation in the brain. Although it is difficult to compare final local concentrations of the two approaches, our results show that we probably remain well within the “safe” range regarding the induction of immune responses. Nevertheless, this indicates that side effects of AON treatment may also depend on design, dose, frequency or delivery of treatment and one should be aware of potential risks [7].

Before AONs can exert their effect, it is crucial that they cross the cell membrane and the nuclear membrane, since splicing takes place in the nucleus [36]. How AONs are taken up and how they are transported to the nucleus is not known. It has been shown in models of DMD that because of the lack of dystrophin protein, affected muscle cells can more easily take up AONs due to the altered properties of their muscle fiber membranes and a more open endothelium [37]. However, mechanisms of AON uptake by intact neurons in the CNS are probably different and may involve utilization of trafficking pathways for cellular uptake of AONs including absorptive endocytosis, pinocytosis and clathrin-, caveolin-, actin-, dynamin- dependent and -independent pathways [36,38-40]. Moreover, AON cellular uptake may exploit the natural pathways of cell-to-cell nucleic acid transportation that may be also involved in micro-RNA transportation [41]. It is very likely that different physical and chemical properties of AONs depending on their chemistry, 2-O’-modifications and length may also be determining factors for the manner and efficiency of uptake [42]. The AON chemistry used in the current study has been shown to be advantageous for nuclear uptake [43].

We also showed that cells of interest in the CeA can take up AONs; NeuN and CRH positive cells represent neurons and cells expressing CRH, a hormone crucial for fear conditioning and orchestration of stress responses in the brain [44], and a putative target of SRC-1 mediated regulation [45]. NeuN positive cells account for the majority of cells taking up AONs. Moreover, we observed sporadic AON uptake by astrocytes and little or no by microglia. The low uptake by microglia cells may be due to either the properties of those cells, or the fact that they seem to arrive at the injection site probably after AONs have been already taken up by other cells. The fraction of AON-positive astrocytes was substantially lower than for NeuN-positive cells. Other studies suggested that in primates AON uptake by astrocytes may be more substantial [21]. On the other hand, GFAP staining visualizes only part of the total population of astrocytes [46] since some astrocytes do not express GFAP [47]. Hence, it is possible that GFAP negative astrocytes may have taken up AONs. To summarize, based on our findings we can conclude that generally neurons in the brain take up AONs, without, however, being able to rule out the possibility that different populations of neurons may display uptake at different rates, efficiencies or even complete lack of AON uptake. In the injected areas in the CeA, though, the vast majority of NeuN positive cells take up AONs.

It is important to mention that we did not detect the AONs directly, but rather the fluorophore with which they were labeled. Since this can be cleaved off, it would be possible that we detected fluorophores that were not bound to the AONs. However, that is not likely since uptake takes place very rapidly after injections, when little or no degradation of the AON-fluorophore complex is expected. Moreover, the considerable effect on exon skipping 3 and 7 days after an injection indicates AON activity which coincides with detection of fluorescence in the cells. For this study we made the assumption of equal stability between the two AONs.

Although the addition of a fluorophore increases hydrophobicity, hence cellular trafficking, it also increases its size. Therefore, the diffusion we observe here might be an over- or an underestimation of what it would be without the fluorophore attached. Importantly, efficacy has been shown to be similar between labeled and unlabeled AONs [48,49]. Finally, our measurements of the diffusion of the AONs indicate that a specific brain region can be targeted with minimal leakage to adjacent areas. The diffusion observed here is likely a function of the targeted area, the volume and AON concentration and the injection rate and it may not be possible to directly extrapolate to other situations. Nevertheless, one would assume that with an optimal combination of volume and concentration smaller regions may also be targeted with reasonable specificity.

## Conclusions

In conclusion, we have shown that it is possible to induce specific exon skipping and subsequent isoform switching of SRC-1 in the CeA without noticeable adverse effects. Our future work will address the functional consequences of SRC-1 isoform switching, as well as the many additional genes that are potential targets of such. This use of isoform switching with AONs has great potential that it must be considered not only in cases where it can restore aberrant gene expression and function, but also as an important molecular tool for manipulation of gene expression that constitutes an alternative to RNA interference or knock-out models.

## Methods

### Animals, stereotactic surgery and tissue processing

C57bl/6j male mice between the ages of 12-14 weeks (Janvier SAS, France) were used for all experiments. Animals were singly housed in individually ventilated cages at a 12 hour light cycle with lights on at 7 am. Food and water were available *ad libitum*. All animal experiments were carried out in accordance with European Communities Council Directive 86/609/EEC and the Dutch law on animal experiments and were approved by the Leiden University animal ethical committee (protocol number: 10128). Animals were anesthetized with a cocktail of Hypnorm-Dormicum-demineralized water in a volume ratio of 1.33:1:3. The depth of anesthesia was always confirmed by examining the paw and tail reflexes. When mice were deeply anesthetized they were mounted on a Kopf stereotact (David Kopf instruments, Tujunga, CA, USA). For every experiment, animals were bilaterally injected with 0.5 μl of the appropriate solution (sterile saline, AONs at a concentration of 400 pmol/μl in sterile saline (Eurogentec, Liège, Belgium)) in the central amygdala (coordinates relative to bregma: -1.25 mm anterior-posterior, ±2.95 mm medio-lateral and -4.75 mm dorso-ventral) [50]. For injections, customized borosilicate glass micro-capillary tips of approximately 100 μm in diameter, connected to a Hamilton needle (5 μl, 30 gauge) were used. The Hamilton syringe was connected to an injection pump (Harvard apparatus, Holliston, MA, USA) which controlled the injection rate set at 0.15 μl/min. After surgery the animals were returned to the home cage and remained undisturbed until sacrifice, with the exception of daily weighing in order to monitor their recovery from surgery. To assess mRNA expression, animals were decapitated after an intraperitoneal injection (i.p.) of overdose Euthasol (ASTfarma, Oudewater, the Netherlands), brains were removed quickly and snap frozen on dry ice. For detection of the presence of AONs over time and putative immunostimulatory effects, mice were sacrificed with transcardial perfusion with a solution of 4% paraformaldehyde (PFA) (Sigma-Aldrich, Zwijndrecht, the Netherlands) in 0.1 M phosphate buffer saline (PBS) after an i.p. injection of overdose Euthasol, 1, 3, 5, 7 and 14 days after the injection. Each time point contained 3-7 animals. In order to assess potential immunostimulatory effects we used animals injected with 0.5 μl sterile saline (vehicle) that were sacrificed either 3 or 7 days (4 per group) after the injection as controls for the respective time points. Three additional animals were sacrificed without having been operated on. After sacrificing the animals, the brains were removed and postfixated overnight in 4% PFA at 4°C. Subsequently they were cryoprotected in 15% and 30% sucrose, snap frozen on dry ice and stored at -80°C.

### Antisense oligonucleotides

Two different green fluorophore labeled AONs were used: one targeting human dystrophin, which has no known targets in the mouse (CGCCGCCAUUUCUCAACAG), labeled with a fluorescein amidite (FAM) fluorophore and one targeting exon 22 of SRC-1, that is specific for the SRC-1e splice variant (CUGUAGUCACCACAGAGAAG), labeled with Alexa Fluor® 488. The AON against exon 22 of SRC-1e was administered in order to investigate whether it can induce exon skipping, whereas the AON against human dystrophin was used as control to study cellular uptake and potential immunostimulatory effects. AONs were modified with a full-length phosphorothioate backbone which increases AON stability and cellular uptake and consisted of 2-O’-methyl RNA to render them RNase H resistant and to counterbalance potential immunostimulatory effects caused by the phosphorothioate modified backbone [34,35,51,52].

### Immunofluorescence

Brains were sectioned at a thickness of 25 μm on a Leica cryostat and sections stored in antifreeze solution [30% ethylene glycol (Merck, Darmstadt, Germany), 20% glycerol (Sigma-Aldrich), 0.02 M Na_2_HPO_4_ (Merck), 6.6 mM NaH_2_PO_4_ (merck)] at -20°C until use. Before use sections were washed in PBS to remove anti-freeze. Subsequently sections were incubated in 0.5% triton X-100 (Sigma-Aldrich) in PBS for 30 min to increase permeability of the cells and washed with PBS. Blocking with 2% normal donkey serum (Brunschwig Chemie, Amsterdam, the Netherlands) in PBS-B^TSA^ for 45 min was followed by an overnight incubation with primary antibody at room temperature (Table 2). Afterwards, the primary antibody was washed out with PBS and incubation with the secondary antibody (Table 2) followed for 2.5-3 h. The secondary antibody was washed followed by 10 min incubation with Hoechst (1:10000) (Hoechst 33258, pentahydrate, bis-benzymide, Invitrogen, Breda, the Netherlands) and another PBS washing step. Finally, the sections were mounted on glass slides, dried and coverslipped with Aqua Polymount (Polysciences Inc, Eppelheim, Germany). Slides were stored at 4°C until observation.

**Table 2:** Antibodies and dilutions used for all immunofluorescent stainings

**Primary antibodies**				**Secondary antibodies**		
**Marker**	**Type**	**Manufacturer**	**Dilution**	**Type**	**Manufacturer**	**Dilution**
CRH	Goat polyclonal	Santa Cruz biotechnology, Heidelberg, Germany	1:250	Donkey anti-goat	Invitrogen, Breda, the Netherlands	1:100
NeuN	Mouse monoclonal	Chemicon, Amsterdam, the Netherlands	1:200	Donkey anti-mouse	Invitrogen, Breda, the Netherlands	1:500
GFAP	Mouse monoclonal	Santa Cruz biotechnology, Heidelberg, Germany	1:1000	Donkey anti-mouse	Invitrogen, Breda, the Netherlands	1:500
IBA-1	Goat monoclonal	Santa Cruz biotechnology, Heidelberg, Germany	1:200	Donkey anti-goat	Invitrogen, Breda, the Netherlands	1:400
CD-45	Rat polyclonal	Serotec, Düsseldorf, Germany	1:1000	Donkey anti-rat	Invitrogen, Breda, the Netherlands	1:500

The fluorophore of all secondary antibodies was Alexa Fluor® 594.

### Microscopy

Confocal imaging was performed on a Nikon Eclipse TE 200-E microscope. Confocal images were collected as z-stacks at a magnification of 200 or 600 times with a z step size of 0.5 μm and an image size of 1024×1024 pixels. When two or more markers were determined in a single section, the different channels were imaged separately to avoid artifacts due to overlap of the emission wavelengths of the fluorescent labels. The same settings were used to obtain images for quantification (e.g. at different time points, between subjects or between groups for the same marker). Z-stacks were converted to .avi format and then stored as single image .tiff files using the z-projection function of Image J (NIH, Bethesda, MD) with standard deviation as projection type. Images of damaged sections or images with artifacts were excluded from further analysis. Finally, to examine cellular uptake and colocalization of different markers we merged different channels of the same image in Image J.

### Image processing

Appropriate thresholds were applied to correct for background. For each marker the positive stained area was presented as a percentage of the total area of the visual field. In order to reduce measurement bias, holes or ruptures in the tissue were not taken into account for the calculation of total area. For determination of immune responses 3-4 pictures were used per brain and the mean value of those was used as the sample value.

### Diffusion of the AONs

For determination of the diffusion of the AONs in the brain we measured the diffusion of the green fluorescence in the medio-lateral axis and the dorso-ventral axis in Image J on 4 or 5 10 μm-thick sections per brain (n=6) which were taken 80 μm apart from each other. Images were taken on a Nikon eclipse 6800 fluorescent microscope at 100X magnification. Before measurements, appropriate background correction was applied. Lines were drawn along the medio-lateral and dorso-ventral axes and their length was measured in pixels. With help of a calibration slide we converted the values from pixels to μm. The positive area for green fluorescence was also measured and total positive volume was calculated according to Cavalieri’s rule. Mean and maximum diffusion distances were calculated as well as total volume per sample.

### Laser microdissection and RNA processing

Cryosections at a thickness of 10 μm were taken from snap frozen brains and mounted on polyethylene naphthalate membrane slides (Carl Zeiss, Munich, Germany). Up to 5 sections were mounted on a slide with adjacent sections being on different slides. The slides were stored at -80°C until laser microdissection. Laser microdissection was carried out on a Palm laser microdissection microscope as has been described elsewhere [53,54]. Briefly, sections were observed under fluorescent light in order to determine regions that had taken up AONs. With the assistance of appropriate software the desired regions were selected, microdissected and collected in adhesive caps (Carl Zeiss). Collected tissue was then stored in Trizol (Invitrogen) at 4°C until RNA isolation, which was always carried out the same day as laser microdissection in order to preserve RNA quality. RNA isolation was performed as has been described elsewhere [55]. Briefly, RNA was isolated with chloroform and precipitated with isopropanol and linear acrylamide. RNA pellets were rinsed with ice cold ethanol 75%, air-dried and resuspended with RNase-free DEPC-treated demineralized water. Quality and concentration of RNA samples were measured on a Bioanalyzer 2100 (Agilent Technologies, Santa Clara, CA, USA) using the RNA 600 Pico LabChip according to the manufacturer’s instructions.

### cDNA synthesis

RNA samples were first treated with DNAse I (Invitrogen) to remove potential genomic DNA contamination. Subsequently, RNA samples were reverse transcribed with iScript cDNA synthesis kit (Bio-Rad, Hercules, CA, USA). Briefly, 4 μl of 5 times iScript reaction mix, 1 μl of iScript reverse transcriptase and 5 μl of Nuclease-free H_2_O were added to 10 μl of DNase I treated RNA. Subsequently samples were incubated for 5 min at 25°C followed by 30 min at 40°C and finally 5 min at 85°C in a PTC-200 DNA engine cycler (Bio-Rad).

### qPCR

Quantitative polymerase chain reaction (qPCR) was performed for assessment of gene expression in the CeA of AON injected mice. A 1:1 dilution of cDNA in autoclaved demineralized water was used for qPCR. The quantification of cDNA was performed on a LightCycler 2.0 (Roche Applied Science, Basel, Switzerland) using LC FastStartDNA Master^PLUS^ SYBR Green I (Roche). 2.5 μl of cDNA was added to a mix of 2 μl 5 times Sybr green mix, 1 μl of both forward and reverse primers (5 μM) and 3.5 μl nuclease-free water, in LightCycler Capillaries (20 μl, Roche). All measurements were performed in duplicate. The PCR program comprised 10 min at 95°C followed by 45 cycles of denaturation at 95°C for 10 sec, annealing at 60°C for 10 sec and elongation at 72°C for 10 sec, with a subsequent dissociation stage (from 65°C to 95°C, at a rate of 0.1°C/sec). The SRC-1 splice variants were quantified as an expression ratio of SRC-1a/SRC-1e; the expression of total SRC-1 and GR was normalized against β-actin. Quantification of relative expression was calculated using the Pfaffl method [56] and normalized against the control group (dystrophin AON).

The forward and reverse primers used for the different genes were respectively: 5^′^-CCTCTACTGCAACCAGCTCTCGTC-3^′^ and 5^′^-TGCTGCACCTGCTGGTTTCCAT-3^′^ for SRC-1a; 5^′^-TGCAACCAGCTCTCGTCCACTG-3^′^ and 5^′^-GCTCCTCTAGTCTGTAGTCACCACA-3^′^ for SRC-1e; 5^′^-CGACCGCAGAGCAGCAGTTA-3^′^ and 5^′^-GCCGCTCAGTCAGAGAGCTG-3^′^ for total SRC-1; 5^′^-CCCTCCCATCTAACCATCCT-3^′^ and 5^′^-ACATAAGCGCCACCTTTCTG-3^′^ for GR; 5^′^-TTTCCCACAGCAGTACGCAT-3^′^ and 5^′^-TAATTTGGCCGCTGTCCCAT-3^′^ for SRC-2; 5^′^-CAACGAGCGGTTCCGATG-3^′^ and 5^′^-GCCACAGGATTCCATACCCA-3^′^ for β-actin.

### Statistical analysis

For comparisons between two groups an independent t-test was used. For comparisons among multiple groups one-way ANOVA was used followed by Tukey’s post-hoc test (for comparison of immunostimulatory effects between all groups) or Dunnett’s post-hoc test (for comparison of fluorescence intensity at different time points with fluorescence intensity after one day post-injection). All data are presented as mean ± SEM.

## Competing interests

Annemieke M. Aartsma-Rus reports being an employee of Leiden University Medical Center and coinventor on patent applications for antisense sequences and exon skipping technology. Leiden University Medical Center has licensed the rights to part of these patents exclusively to Prosensa Therapeutics. The inventors specified on the patents (including Annemieke M. Aartsma-Rus) are jointly entitled to a share of royalties paid to Leiden University Medical Center, should the therapy eventually be brought to the market. The other authors declare absence of any commercial or financial relationships that could be construed as a potential conflict of interest.

## Authors’ contributions

IZ performed stereotactic operations, participated in immunofluorescent stainings, acquisition of confocal images, image processing, qPCR analysis, performed statistical analysis and drafted the manuscript, GG participated in immunofluorescent stainings, image processing and acquisition of confocal images, LTCMvW carried out qPCR analysis, YA participated in laser microdissection and in drafting the manuscript, SRdK participated in immunofluorescent stainings and qPCR analysis, NAD designed the laser microdissection experiments and participated in drafting the manuscript, WMCvR-M designed the study and participated in drafting the manuscript, AMA-R designed the study and participated in drafting the manuscript, OCM conceived of the study and supervised its design and coordination, obtained funding and participated in drafting the manuscript. All authors have read and approved the final manuscript.

## References

[B1] Fagnani M Barash Y Ip JY Misquitta C Pan Q Saltzman AL Shai O Lee L Rozenhek A Mohammad N Functional coordination of alternative splicing in the mammalian central nervous system Genome Biol 2007 8 6 R108 10.1186/gb-2007-8-6-r108 17565696 PMC2394768

[B2] Ule J Ule A Spencer J Williams A Hu JS Cline M Wang H Clark T Fraser C Ruggiu M Nova regulates brain-specific splicing to shape the synapse Nat Genet 2005 37 8 844 852 10.1038/ng1610 16041372

[B3] Vallone D Picetti R Borrelli E Structure and function of dopamine receptors Neurosci Biobehav R 2000 24 1 125 132 10.1016/S0149-7634(99)00063-9 10654668

[B4] Zmijewski MA Slominski AT Modulation of corticotropin releasing factor (CRF) signaling through receptor splicing in mouse pituitary cell line AtT-20–emerging role of soluble isoforms J Physiol Pharmacol 2009 60 Suppl 4 39 46 20083850 PMC2814449

[B5] Ryberg E Vu HK Larsson N Groblewski T Hjorth S Elebring T Sjogren S Greasley PJ Identification and characterisation of a novel splice variant of the human CB1 receptor FEBS Lett 2005 579 1 259 264 10.1016/j.febslet.2004.11.085 15620723

[B6] Liu QR Pan CH Hishimoto A Li CY Xi ZX Llorente-Berzal A Viveros MP Ishiguro H Arinami T Onaivi ES Species differences in cannabinoid receptor 2 (CNR2 gene): identification of novel human and rodent CB2 isoforms, differential tissue expression and regulation by cannabinoid receptor ligands Genes Brain Behav 2009 8 5 519 530 10.1111/j.1601-183X.2009.00498.x 19496827 PMC3389515

[B7] Zalachoras I Evers MM van Roon-Mom WM Aartsma-Rus AM Meijer OC Antisense-mediated RNA targeting: versatile and expedient genetic manipulation in the brain Front Mol Neurosci 2011 4 10 21811437 10.3389/fnmol.2011.00010 PMC3142880

[B8] Evers MM Pepers BA van Deutekom JC Mulders SA den Dunnen JT Aartsma-Rus A van Ommen GJ van Roon-Mom WM Targeting several CAG expansion diseases by a single antisense oligonucleotide PLoS One 2011 6 9 e24308 10.1371/journal.pone.0024308 21909428 PMC3164722

[B9] Yang J Fuller PJ Interactions of the mineralocorticoid receptor – Within and without Mol Cell Endocrinol 2012 350 2 196 205 10.1016/j.mce.2011.07.001 21784126

[B10] Hua Y Sahashi K Hung G Rigo F Passini MA Bennett CF Krainer AR Antisense correction of SMN2 splicing in the CNS rescues necrosis in a type III SMA mouse model Genes Dev 2010 24 15 1634 1644 10.1101/gad.1941310 20624852 PMC2912561

[B11] Zakharov MN Pillai BK Bhasin S Ulloor J Istomin AY Guo C Godzik A Kumar R Jasuja R Dynamics of coregulator-induced conformational perturbations in androgen receptor ligand binding domain Mol Cell Endocrinol 2011 341 1–2 1 8 21605623 10.1016/j.mce.2011.03.003

[B12] Dobrovolna J Chinenov Y Kennedy MA Liu B Rogatsky I Glucocorticoid-dependent phosphorylation of the transcriptional coregulator GRIP1 Mol Cell Biol 2012 32 4 730 739 10.1128/MCB.06473-11 22158970 PMC3272970

[B13] Goemans NM Tulinius M van den Akker JT Burm BE Ekhart PF Heuvelmans N Holling T Janson AA Platenburg GJ Sipkens JA Systemic administration of PRO051 in Duchenne's muscular dystrophy N Engl J Med 2011 364 16 1513 1522 10.1056/NEJMoa1011367 21428760

[B14] Biddie Simon C John S Sabo Pete J Thurman Robert E Johnson Thomas A Schiltz RL Miranda Tina B Sung M-H Trump S Lightman Stafford L Transcription factor AP1 potentiates chromatin accessibility and glucocorticoid receptor binding Mol Cell 2011 43 1 145 155 10.1016/j.molcel.2011.06.016 21726817 PMC3138120

[B15] Williams JH Schray RC Patterson CA Ayitey SO Tallent MK Lutz GJ Oligonucleotide-mediated survival of motor neuron protein expression in CNS improves phenotype in a mouse model of spinal muscular atrophy J Neurosci 2009 29 24 7633 7638 10.1523/JNEUROSCI.0950-09.2009 19535574 PMC6665627

[B16] Burghes AH McGovern VL Antisense oligonucleotides and spinal muscular atrophy: skipping along Genes Dev 2010 24 15 1574 1579 10.1101/gad.1961710 20679391 PMC2912553

[B17] Nlend Nlend R Meyer K Schumperli D Repair of pre-mRNA splicing: prospects for a therapy for spinal muscular atrophy RNA Biol 2010 7 4 430 440 10.4161/rna.7.4.12206 20523126 PMC3070909

[B18] Pitts MW Todorovic C Blank T Takahashi LK The central nucleus of the amygdala and corticotropin-releasing factor: insights into contextual fear memory J Neurosci 2009 29 22 7379 7388 10.1523/JNEUROSCI.0740-09.2009 19494159 PMC2771694

[B19] Pitts MW Takahashi LK The central amygdala nucleus via corticotropin-releasing factor is necessary for time-limited consolidation processing but not storage of contextual fear memory Neurobiol Learn Mem 2011 95 1 86 91 10.1016/j.nlm.2010.11.006 21093597 PMC3022075

[B20] Ma L Wang D-D Zhang T-Y Yu H Wang Y Huang S-H Lee FS Chen Z-Y Region-specific involvement of BDNF secretion and synthesis in conditioned taste aversion memory formation J Neurosci 2011 31 6 2079 2090 10.1523/JNEUROSCI.5348-10.2011 21307245 PMC3044502

[B21] Smith RA Miller TM Yamanaka K Monia BP Condon TP Hung G Lobsiger CS Ward CM McAlonis-Downes M Wei H Antisense oligonucleotide therapy for neurodegenerative disease J Clin Invest 2006 116 8 2290 2296 10.1172/JCI25424 16878173 PMC1518790

[B22] Kamei Y Xu L Heinzel T Torchia J Kurokawa R Gloss B Lin SC Heyman RA Rose DW Glass CK A CBP integrator complex mediates transcriptional activation and AP-1 inhibition by nuclear receptors Cell 1996 85 3 403 414 10.1016/S0092-8674(00)81118-6 8616895

[B23] Kalkhoven E Valentine JE Heery DM Parker MG Isoforms of steroid receptor co-activator 1 differ in their ability to potentiate transcription by the oestrogen receptor EMBO J 1998 17 1 232 243 10.1093/emboj/17.1.232 9427757 PMC1170374

[B24] Meijer OC Kalkhoven E van der Laan S Steenbergen PJ Houtman SH Dijkmans TF Pearce D de Kloet ER Steroid receptor coactivator-1 splice variants differentially affect corticosteroid receptor signaling Endocrinology 2005 146 3 1438 1448 15564339 10.1210/en.2004-0411

[B25] Tetel MJ Auger AP Charlier TD Who's in charge? Nuclear receptor coactivator and corepressor function in brain and behavior Front Neuroendocrinol 2009 30 3 328 342 10.1016/j.yfrne.2009.04.008 19401208 PMC2720417

[B26] Meijer OC Steenbergen PJ De Kloet ER Differential expression and regional distribution of steroid receptor coactivators SRC-1 and SRC-2 in brain and pituitary Endocrinology 2000 141 6 2192 2199 10.1210/en.141.6.2192 10830308

[B27] van der Laan S Lachize SB Vreugdenhil E de Kloet ER Meijer OC Nuclear receptor coregulators differentially modulate induction and glucocorticoid receptor-mediated repression of the corticotropin-releasing hormone gene Endocrinology 2008 149 2 725 732 18006628 10.1210/en.2007-1234

[B28] Tynan RJ Naicker S Hinwood M Nalivaiko E Buller KM Pow DV Day TA Walker FR Chronic stress alters the density and morphology of microglia in a subset of stress-responsive brain regions Brain Behav Immun 2010 24 7 1058 1068 10.1016/j.bbi.2010.02.001 20153418

[B29] Herber DL Maloney JL Roth LM Freeman MJ Morgan D Gordon MN Diverse microglial responses after intrahippocampal administration of lipopolysaccharide Glia 2006 53 4 382 391 10.1002/glia.20272 16288481

[B30] Apostolakis EM Ramamurphy M Zhou D Oñate S O’Malley BW Acute disruption of select steroid receptor coactivators prevents reproductive behavior in rats and unmasks genetic adaptation in knockout mice Mol Endocrinol 2002 16 7 1511 1523 10.1210/me.16.7.1511 12089347

[B31] Winkler J Stessl M Amartey J Noe CR Off-target effects related to the phosphorothioate modification of nucleic acids Chem Med Chem 2010 5 8 1344 1352 20544786 10.1002/cmdc.201000156

[B32] Jeanneteau FD Lambert WM Ismaili N Bath KG Lee FS Garabedian MJ Chao MV BDNF and glucocorticoids regulate corticotrophin-releasing hormone (CRH) homeostasis in the hypothalamus Proc Natl Acad Sci 2012 109 4 1305 1310 10.1073/pnas.1114122109 22232675 PMC3268297

[B33] Chiasson BJ Armstrong JN Hooper ML Murphy PR Robertson HA The application of antisense oligonucleotide technology to the brain: some pitfalls Cell Mol Neurobiol 1994 14 5 507 521 10.1007/BF02088834 7621510 PMC11566955

[B34] Okun E Lathia JD Mattson MP Adhesion- and migration-related side effects of phosphothioated CpG oligodeoxynucleotides Cell Adh Migr 2009 3 3 272 274 10.4161/cam.3.3.8692 19458479 PMC2712808

[B35] Robbins M Judge A Liang L McClintock K Yaworski E MacLachlan I 2'-O-methyl-modified RNAs act as TLR7 antagonists Mol Ther 2007 15 9 1663 1669 10.1038/sj.mt.6300240 17579574

[B36] Juliano RL Ming X Nakagawa O Cellular uptake and intracellular trafficking of antisense and siRNA oligonucleotides Bioconjug Chem 2012 23 2 147 157 10.1021/bc200377d 21992697 PMC3288458

[B37] Heemskerk H de Winter C van Kuik P Heuvelmans N Sabatelli P Rimessi P Braghetta P van Ommen GJ de Kimpe S Ferlini A Preclinical PK and PD studies on 2'-O-methyl-phosphorothioate RNA antisense oligonucleotides in the mdx mouse model Mol Ther 2010 18 6 1210 1217 10.1038/mt.2010.72 20407428 PMC2889733

[B38] Akhtar S Juliano RL Cellular uptake and intracellular fate of antisense oligonucleotides Trends Cell Biol 1992 2 5 139 144 10.1016/0962-8924(92)90100-2 14731968

[B39] Alam MR Ming X Dixit V Fisher M Chen X Juliano RL The biological effect of an antisense oligonucleotide depends on its route of endocytosis and trafficking Oligonucleotides 2010 20 2 103 109 10.1089/oli.2009.0211 20038250 PMC2883474

[B40] Koller E Vincent TM Chappell A De S Manoharan M Bennett CF Mechanisms of single-stranded phosphorothioate modified antisense oligonucleotide accumulation in hepatocytes Nucleic Acids Res 2011 39 11 4795 4807 10.1093/nar/gkr089 21345934 PMC3113586

[B41] Kosaka N Iguchi H Yoshioka Y Takeshita F Matsuki Y Ochiya T Secretory mechanisms and intercellular transfer of microRNAs in living cells J Biol Chem 2010 285 23 17442 17452 10.1074/jbc.M110.107821 20353945 PMC2878508

[B42] Manoharan M Johnson LK McGee DPC Guinosso CJ Ramasamy K Springer RH Bennett CF Ecker DJ Vickers T Cowsert L Chemical modifications to improve uptake and bioavailability of antisense oligonucleotides Ann N Y Acad Sci 1992 660 1 306 309 10.1111/j.1749-6632.1992.tb21095.x 1364095

[B43] Aartsma-Rus A Kaman WE Bremmer-Bout M Janson AA den Dunnen JT van Ommen GJ van Deutekom JC Comparative analysis of antisense oligonucleotide analogs for targeted DMD exon 46 skipping in muscle cells Gene Ther 2004 11 18 1391 1398 10.1038/sj.gt.3302313 15229633

[B44] Kolber BJ Roberts MS Howell MP Wozniak DF Sands MS Muglia LJ Central amygdala glucocorticoid receptor action promotes fear-associated CRH activation and conditioning Proc Natl Acad Sci USA 2008 105 33 12004 12009 10.1073/pnas.0803216105 18695245 PMC2575312

[B45] Lachize S Apostolakis EM van der Laan S Tijssen AM Xu J de Kloet ER Meijer OC Steroid receptor coactivator-1 is necessary for regulation of corticotropin-releasing hormone by chronic stress and glucocorticoids Proc Natl Acad Sci USA 2009 106 19 8038 8042 10.1073/pnas.0812062106 19416907 PMC2683087

[B46] Bushong EA Martone ME Jones YZ Ellisman MH Protoplasmic astrocytes in CA1 stratum radiatum occupy separate anatomical domains J Neurosci 2002 22 1 183 192 11756501 10.1523/JNEUROSCI.22-01-00183.2002 PMC6757596

[B47] Takeichi T Takarada-Iemata M Hashida K Sudo H Okuda T Kokame K Hatano T Takanashi M Funabe S Hattori N The effect of Ndrg2 expression on astroglial activation Neurochem Int 2011 59 1 21 27 10.1016/j.neuint.2011.03.019 21672576

[B48] Aartsma-Rus A Janson AAM Kaman WE Bremmer-Bout M den Dunnen JT Baas F van Ommen G-JB van Deutekom JCT Therapeutic antisense-induced exon skipping in cultured muscle cells from six different DMD patients Hum Mol Genet 2003 12 8 907 914 10.1093/hmg/ddg100 12668614

[B49] Aartsma-Rus A Janson AAM Kaman WE Bremmer-Bout M van Ommen G-JB den Dunnen JT van Deutekom JCT Antisense-induced multiexon skipping for duchenne muscular dystrophy makes more sense Am J Hum Genet 2004 74 1 83 92 10.1086/381039 14681829 PMC1181915

[B50] Paxinos G Franklin KBJ The mouse brain in stereotaxic coordinates 2001 USA: Academic Press, Elsevier

[B51] Sioud M Furset G Cekaite L Suppression of immunostimulatory siRNA-driven innate immune activation by 2'-modified RNAs Biochem Biophys Res Commun 2007 361 1 122 126 10.1016/j.bbrc.2007.06.177 17658482

[B52] Hamm S Latz E Hangel D Müller T Yu P Golenbock D Sparwasser T Wagner H Bauer S Alternating 2'-O-ribose methylation is a universal approach for generating non-stimulatory siRNA by acting as TLR7 antagonist Immunobiology 2010 215 7 559 569 10.1016/j.imbio.2009.09.003 19854535

[B53] Datson NA Meijer L Steenbergen PJ Morsink MC van der Laan S Meijer OC de Kloet ER Expression profiling in laser-microdissected hippocampal subregions in rat brain reveals large subregion-specific differences in expression Eur J Neurosci 2004 20 10 2541 2554 10.1111/j.1460-9568.2004.03738.x 15548198

[B54] Erickson HS Albert PS Gillespie JW Rodriguez-Canales J Marston Linehan W Pinto PA Chuaqui RF Emmert-Buck MR Quantitative RT-PCR gene expression analysis of laser microdissected tissue samples Nat Protoc 2009 4 6 902 922 10.1038/nprot.2009.61 19478806 PMC2760821

[B55] Datson NA Morsink MC Steenbergen PJ Aubert Y Schlumbohm C Fuchs E de Kloet ER A molecular blueprint of gene expression in hippocampal subregions CA1, CA3, and DG is conserved in the brain of the common marmoset Hippocampus 2009 19 8 739 752 10.1002/hipo.20555 19156849

[B56] Pfaffl MW A new mathematical model for relative quantification in real-time RT-PCR Nucleic Acids Res 2001 29 9 e45 10.1093/nar/29.9.e45 11328886 PMC55695

